# Development and Application of an UPLC–MS/MS Method for Simultaneous Quantification of Abemaciclib and Tamoxifen with Their Active Metabolites in Rat Plasma: Application to a Pharmacokinetic Study

**DOI:** 10.3390/ph19050795

**Published:** 2026-05-19

**Authors:** Yahya Alshehri, Abdulrhman Al-Majed, Ahmad Obaidullah, Yousef Bin Jardan, Ahmed Bakheit, Mohamed Hefnawy

**Affiliations:** 1 Department of Pharmaceutical Chemistry, College of Pharmacy, King Saud University, Riyadh 11451, Saudi Arabia; yalshehri20@gmail.com (Y.A.); aalmajed@ksu.edu.sa (A.A.-M.); aobaidullah@ksu.edu.sa (A.O.); abakheit@ksu.edu.sa (A.B.); 2 Department of Pharmaceutics, College of Pharmacy, King Saud University, Riyadh 11451, Saudi Arabia; ybinjardan@ksu.edu.sa

**Keywords:** UPLC–MS/MS, breast cancer, abemaciclib, tamoxifen, metabolites, rat plasma, pharmacokinetics

## Abstract

**Background:** Abemaciclib (ABM) in combination with tamoxifen (TAM) is an extremely significant treatment regimen for hormone receptor-positive (HR+), human epidermal growth factor receptor 2-negative (HER2-) breast cancer. It is approved for patients to reduce the risk of cancer recurrence. A bioanalytical method for the simultaneous determination of this new anti-breast cancer combination and its pharmacokinetic application has not yet been reported. **Methods**: An ultra-performance liquid chromatography tandem mass spectrometry (UPLC–MS/MS) method was developed for quantifying ABM, TAM, and its metabolites, including abemaciclib active metabolites M2, M18, and M20 and tamoxifen active metabolite N-desmethyl tamoxifen (NDTAM), in rat plasma using econazole as the internal standard (IS). Chromatographic separation was achieved on a Kinetex C18 column (100 × 2.1 mm ID, 2.6 µm) using gradient elution with 5 mM ammonium formate in water (eluent A) and 5 mM ammonium formate in water/methanol (1:9, *v*/*v*, eluent B) at a flow rate of 0.4 mL/min. Detection was performed on a TSQ Fortis Plus mass spectrometer employing multiple reaction monitoring mode under positive electrospray ionization. **Results**: The developed method was validated according to the guidance of the FDA. Linearity in rat plasma (ng/mL) was achieved from 1 to 1000 for ABM, TAM, and M20; 3 to 1000 for M2; 5 to 500 for M18; and 1 to 500 for NDTAM; with correlation coefficients ranging from 0.9991 to 0.9931 for all analytes using a weighting factor of 1/X^2^. The lower limit of detection (LLOD) ranged between 0.3 and 1.5 ng/mL for all drugs. The accuracy ranged from 96 to 108% and the precision was less than 7.6% RSD for all analytes. For the first time, the newly developed approach was effectively used in a pharmacokinetic study on the simultaneous oral administration of ABM and TAM in rats that received 30.0 mg/kg of ABM and 8.0 mg/kg of TAM. **Conclusions**: To the best of our knowledge, this is the first reported UPLC–MS/MS method for the assay of ABM, TAM, and its active metabolites in plasma. This method offers a bioanalytical tool for assessing the pharmacokinetics of ABM and TAM. Therefore, this study makes a definite significant contribution to the field of bioanalytical research. Further validation in human plasma is required for future clinical or therapeutic drug monitoring applications, as the approach was developed in an animal model.

## 1. Introduction

Breast cancer is one of the most diagnosed cancers in women. It affects different parts of a woman’s breast, including glands, ducts, and fatty tissue [1]. Regrettably, it is obvious that breast cancer is the second leading cause of cancer death in women worldwide after lung cancer and the leading cause of cancer death in Black and Hispanic women [2]. An epidemiological study stated that breast cancer in Saudi Arabia comprised 19.8% of all cancer cases identified in the Kingdom [3]. Chemotherapy, hormonal therapy, and targeted therapy are the three types of systemic treatment used for the treatment or prevention of breast cancer. Drugs from different classes are widely used in combination or as monotherapies based on the cancer stage and the goal of the therapy [4].

Abemaciclib (Verzenio™) is an orally administered third cyclin-dependent kinases 4 and 6 inhibitor developed by Eli Lilly and Company that received FDA approval in 2017 for the treatment of various stages of breast cancer as a monotherapy and in combination with fulvestrant [5]. In October 2021, the FDA granted the approval of abemaciclib in combination with endocrine therapy as an adjuvant treatment for patients with hormone receptor (HR)-positive, human epidermal growth factor receptor 2 (HER2)-negative, node-positive, early breast cancer at high risk of recurrence and a Ki-67 score ≥ 20% [6]. In addition, tamoxifen has long been considered the gold standard for the endocrine treatment of estrogen-receptor-positive breast cancer, and the World Health Organization (WHO) considers it an essential drug for the treatment of breast cancer [7]. Indeed, combination therapy of abemaciclib with tamoxifen provides promising results for the treatment of breast cancer, as mentioned in the FDA approval summary [6].

**Figure 1:**
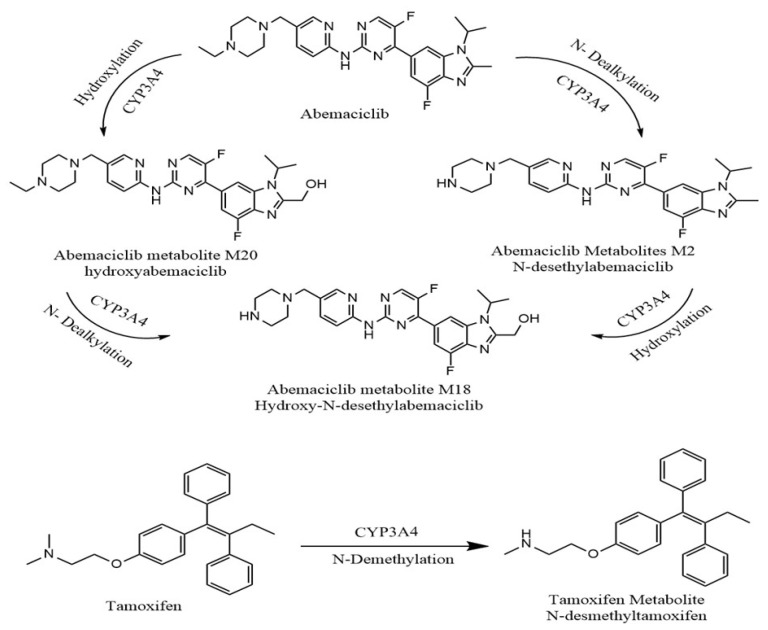
Pathways of abemaciclib (ABM) and tamoxifen (TAM) metabolism by CYP3A4 into their major active metabolites, forming N-desethylabemaciclib (M2), hydroxyabemaciclib (M20), hydroxy-N-desethylabemaciclib (M18), and N-desmethyltamoxifen (NDTAM).

The main route of abemaciclib clearance is hepatic metabolism. Cytochrome P450 (CYP) 3A4 is the primary enzyme responsible for the metabolism of abemaciclib, producing a number of metabolites. The main metabolic route is represented by the synthesis of N-desethylabemaciclib (M2), while hydroxyabemaciclib (M20), hydroxy-N-desethylabemaciclib (M18), and an oxidative metabolite (M1) are other metabolites. However, M2, M18, and M20 have equivalent potency as abemaciclib and their AUCs are 25%, 13%, and 26% of the total circulating analytes in plasma, respectively, versus 34% AUC for abemaciclib [8,9]. The FDA guidelines on safety testing of drug metabolites state that metabolites with greater than 10% of total related drug exposure should be further considered [10]. On the other hand, tamoxifen is significantly biotransformed after oral administration. N-desmethyl tamoxifen is the major circulatory metabolite found in patients’ plasma. N-desmethyl tamoxifen exhibits a similar biological activity as tamoxifen. The metabolic pathways of ABM and TAM are detailed in Figure 1 [11].

The bioanalytical UPLC–MS/MS method developed in this work is the first one reported for the simultaneous quantification of ABM and TAM with their major metabolites, M2, M18, M20, and NDTAM, in rat plasma with pharmacokinetic evaluation. We developed an UPLC–MS/MS method with streamlined sample preparation, rapid analysis, high precision, and a broad linear range for the simultaneous quantification of ABM, TAM, M2, M18, M20, and NDTAM in rat plasma. This is in opposition to previously published bioanalytical methods that either did not include all analytes [12,13,14,15,16,17,18,19,20,21], exhibited low extraction recoveries from biological matrices [19], or did not report pharmacokinetic characterizations [17]. Martínez-Chávez et al. [12] published an LC–MS/MS method but limited it to only four analytes of interest, ABM, M2, M18, and M20, and used an isotopically labelled IS, acknowledging that while this approach represents analytical best practice, it is associated with higher costs and may not be feasible or available in all clinical or resource-limited bioanalytical laboratories. Moreover, Martínez-Chávez et al. [13] and Turković et al. [16] reported an LC–MS/MS method for bioanalytical application but limited it to only ABM with other antineoplastic agents. Recently, Jacobs et al. [22] developed an LC–HRMS method for the determination of eight anti-breast cancer combination drugs that have not received FDA approval, including ABM and TAM but without their metabolites, in four biological matrices. This method used a lot of biological samples and did not offer pharmacokinetic characterization. Collectively, these limitations restrict the throughput of bioanalytical workflows.

Compared to these existing methods, our method offers the advantage of simultaneously detecting ABM and TAM and its metabolites M2, M18, M20, and NDTAM in rat plasma. The method demonstrates excellent precision, accuracy, consistent recoveries, and negligible matrix effects. Additionally, the method incorporates a streamlined sample preparation workflow, rapid chromatographic separation, and minimal solvent consumption. These features facilitate high-throughput analysis of biological samples and enhance its suitability for pharmacokinetic studies.

## 2. Results and Discussion

### 2.1. Method Development and Optimization

**Figure 2:**
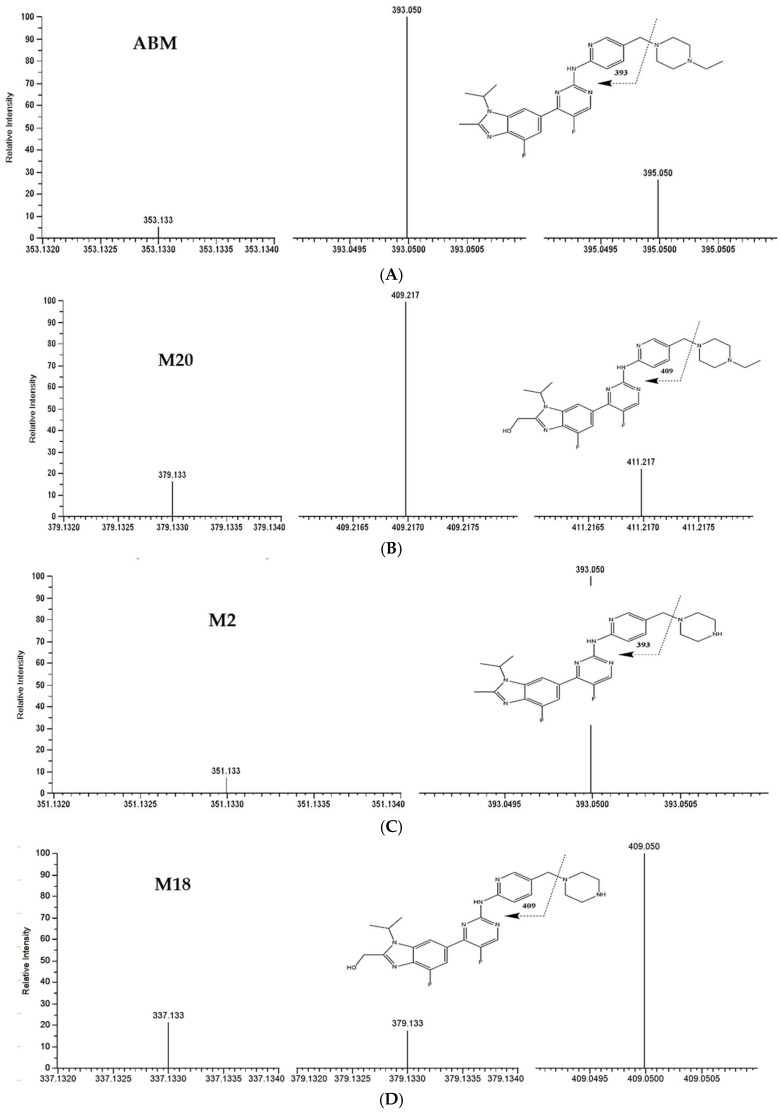

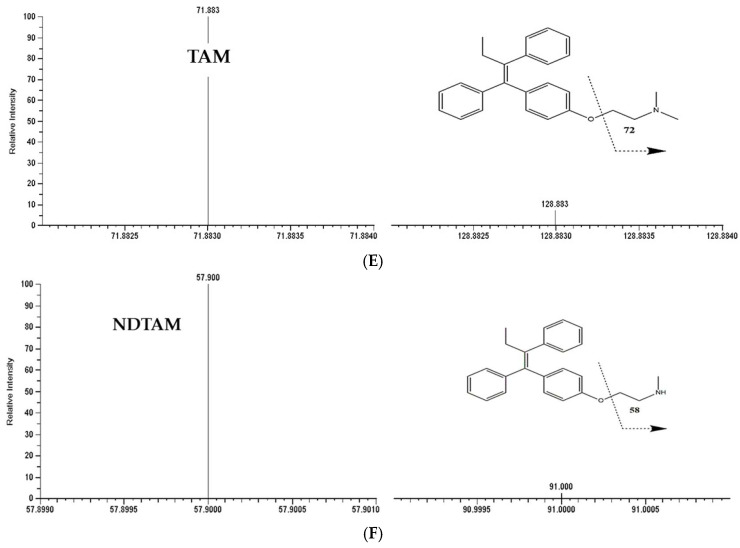
Product ion mass spectra and proposed MS fragmentation of abemaciclib (**A**), M20 (**B**), M2 (**C**), M18 (**D**), tamoxifen (TAM) (**E**), and NDTAM (**F**).

Initially, each analyte targeted in this method (including IS) was analyzed directly via mass spectrometry as a solution containing 1 µg/mL in methanol, as recommended by the manufacturer. All compounds were found to exhibit the most abundant signal in positive ionization mode. The flow rate of infusion was 10 µL/min. The precursor ions were selected for each compound as the single protonation (M + H), while the most abundant product ions were chosen after different mass parameters were tested (Figure 2).

The ion source parameters are quite crucial to optimize, as the optimum condition for one analyte might be problematic for others. However, optimization by selecting the most adequate approach was adopted in this study. Using a continuous flow infusion, the ion source parameters were investigated. A spray voltage of 3800 V was adequate for all analytes. The temperature of the ion source had a significant impact on the signal; however, temperatures of 375 and 400 °C were optimum for the majority of the analytes. The sweep gas had a negative impact on the signal of the analytes; therefore, it was removed and adjusted to zero, while the sheath gas was 45 Arb and the auxiliary gas was 5 Arb. For these optimized parameters, it was found that TAM and NDTAM were the highest signals while M18 and M2 were the lowest.

For the best chromatographic separation, various stationary phases, polar and non-polar, were tested using different column packs that were either cyano-, phenyl-, or octyl (C8) and octadecyl (C18), with varying diameters. The mobile phase consisted of a gradient elution containing 0.1% formic acid in methanol, acetonitrile, and water at multiple ratios. A Kinetex C18 (100 mm × 2.6 µm, 2.1 mm, Phenomenex, Torrance, CA, USA) was superior to the other columns in terms of peak shapes and resolution of the analytes. Nevertheless, methanol was chosen as the organic modifier, which offered much better peak shapes specifically for ABM, which yielded a much wider and tailed peak with acetonitrile [12,13]. During the optimization of the gradient program of the mobile phase, it was found that 40% methanol in the initial condition was optimum for resolving the majority of the analytes, while a fast increase in the organic phase (methanol) up to 90% in less than 1 min caused a faster elution of the later analytes, TAM and NDTAM, without significantly impacting the resolution of the earlier analytes.

**Figure 3:**
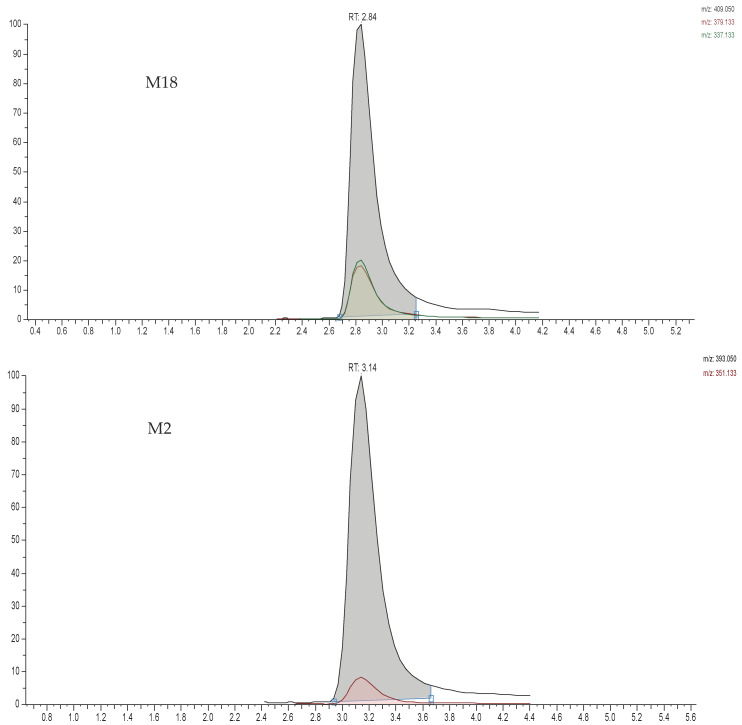

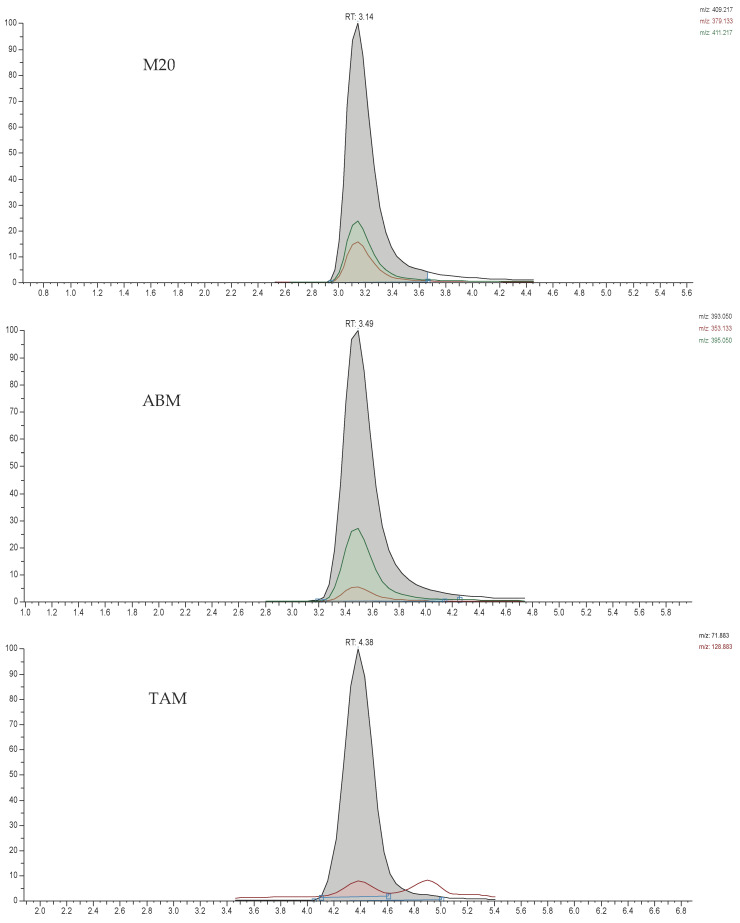

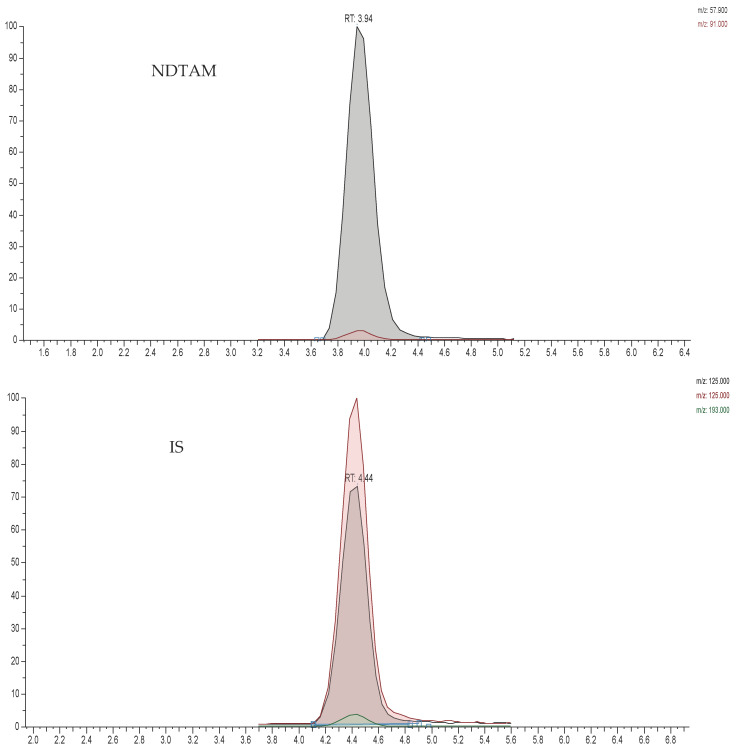
The optimized chromatographic separations for ABM, TAM, their major active metabolites, and IS in rat plasma. The elution sequence was M18 (2.84), M2 (3.14), M20 (3.14), ABM (3.49), NDTAM (3.94), TAM (4.38), and IS (4.44). The green color (small peak) is qualifier ion (*m*/*z*) and the red color (large peak) is quantifier ion (*m*/*z*).

However, the ABM drug and M20, M2, and M18 metabolites still co-eluted. We attempted to separate these analytes from one another, despite the fact that these analytes have different SRM transitions. It was noted that the addition of ammonium formate buffer to the mobile phase offered a much better resolution of the analytes and caused ABM to retain longer than its metabolites. The buffer concentrations were tested at 2.5, 5 and 10 mM ammonium formate. It was found that there was no significant difference between 5 and 10 mM, while 2.5 mM was not adequate for the resolution of the analytes. Therefore, 5 mM (pH ~ 6.8) was the optimum buffer concentration for the mobile phase and it was added at equal concentrations in both mobile phases A and B. Furthermore, we investigated the use of several internal standards, including fluoconazole, econazole, encorafenib, and nateglinide. Econazole was chosen as an internal standard because of its stable ionization, equivalent extraction recovery, lack of considerable endogenous interference, and lack of cross-talk with the MRM channels of abemaciclib, tamoxifen, or their metabolites. Sample treatment via liquid–liquid extraction, protein precipitation, and solid-phase extraction were attempted. It was obvious that protein precipitation using organic solvent was adequate for sample treatment. For that purpose, methanol, acetonitrile, and trichloroacetic acid were tested for protein precipitation, and acetonitrile was far superior for protein precipitation and the recovery of all analytes from the plasma sample, as shown in the results section. A washing solvent using methanol/water (1:1) was used in all sample analysis, methods development, and validation, and it was adequate for that purpose. The optimum chromatographic separations of all studied drugs, their metabolites, and IS from plasma samples are shown in Figure 3.

### 2.2. In Study Validation

#### 2.2.1. Specificity and Selectivity

**Figure 4:**
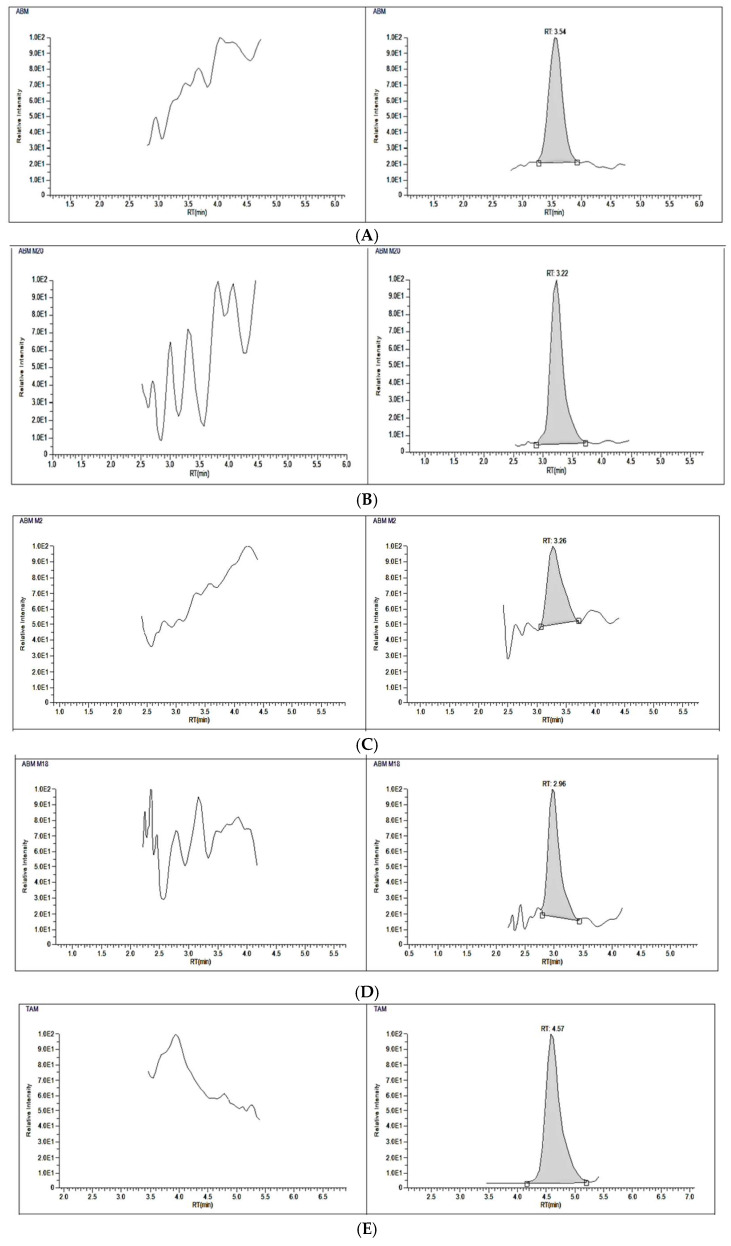

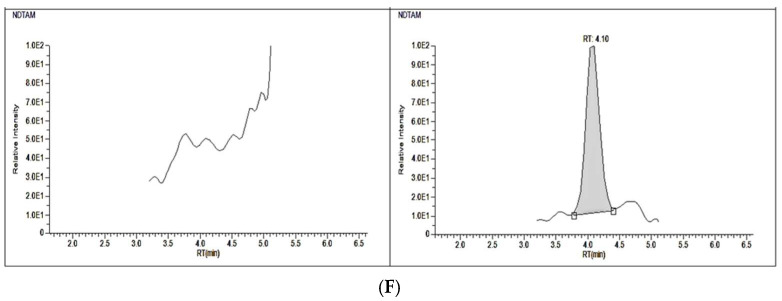
Representative total ion chromatograms for blank rat plasma and rat plasma spiked with LLOQ of abemaciclib (**A**), M20 (**B**), M2 (**C**), M18 (**D**), tamoxifen (TAM) (**E**), and NDTAM (**F**).

The developed LC–MS/MS method was successfully used for the analysis of different lots of plasma samples. First, blank plasma samples were analyzed after multiple cycles of washing solution. Second, plasma samples containing spiked concentrations of ABM, M20, M2, M18, TAM, and NDTAM at LLOQ levels were analyzed. Finally, plasma samples spiked with IS solution were analyzed. No significant interferences were observed at similar retention times above 20% of the response of LLOQ for individual analytes of interest and not more than 5% of the IS response [23]. Representative chromatograms for pretreated blank plasma are shown in Figure 4.

#### 2.2.2. Calibration Curves

The calibration curves were constructed by plotting the peak area ratio of D/IS on the y-axis against the analyte concentration in ng/mL on the x-axis. Linearity in rat plasma (ng/mL) was achieved from 1 to 1000 for ABM, TAM and M20; 3 to 1000 for M2; 5 to 500 for M18; and 1 to 500 for NDTAM. The regression model was utilized with a weighting factor of 1/x^2^, in which x is the analyte concentration. This regression model was convenient for all analytes and yielded a correlation coefficient > 0.99 for all analytes [Table 1]. However, using the regression model without a weighting factor was attempted and the response was linear for all analytes; nevertheless, the accuracy at low levels was dramatically affected. In addition, the calculated concentration of each calibration levels met the required criteria, which were not less than 75% of the calculated results between 85 and 115% and RSD < 15% of the nominal concentration and between 80 and 120% for LLOQ RSD < 20% [23]. More details of the regression parameters are described in Table 1.

#### 2.2.3. Lower Limit of Quantification

The developed method offers good sensitivity for the targeted drugs and their major metabolites. The signal to noise ratio was >20 for all analytes except M2 and M18, which were 12 and 15, respectively. The validated accuracy and precision results of the LLOQ are detailed in Table 2. Furthermore, a representative chromatogram for each compound at LLOQ level is illustrated in Figure 4.

#### 2.2.4. Accuracy and Precision

The accuracy and precision of the intra-assay as well as the inter- assay were assessed using four concentrations of quality control samples: LLOQ, QCL, QCM, and QCH in six duplicates. The determined accuracy and precision are shown in Table 2. The values indicate the average of the measured concentrations, whereas CV is the RSD of these measurements. The values met the acceptance criteria of the guidelines: LLOQ within 20% and the other QCs within 15% [23].

#### 2.2.5. Matrix Effect and Recovery

The extraction recovery and matrix effect were calculated for the measured QC samples level: LQC and HQC. The data are summarized in Table 3. The RSD was ≤15% for all of the studied analytes and met the established criteria in this study [23]. In addition, using a simple protein precipitation as sample pretreatment was adequate to recover all the analytes above >90% within RSD < 10%.

#### 2.2.6. Carry-Over

Carry-over was detected for all analytes, especially for the parent drugs (ABM and TAM) after injection of high concentration of the analytes. The carry-over was less problematic for the metabolites. However, several strategies were conducted to reduce the carry-over to an acceptable level, as per the guidelines. First, a gradient washing step was added after the elution of all analytes and the mobile phase composition returned to the initial conditions. This approach has been utilized in some reports [11,12,13,14]. Second, the extracted samples were diluted in alkaline buffer (mobile phase initial composition), and this step showed the additional elimination of carry-over for all analytes, especially for ABM. This strategy was conducted previously in a report [11]. Finally, the samples were injected into the instrument in an ascending pattern (as expected from low concentration to high concentration) and blanks were injected between high-concentration samples and low-concentration samples [13]. In addition, the IS carry-over was much lower than 5%, which met the established criteria [23].

#### 2.2.7. Dilution Integrity

The spiked plasma at levels above the ULOQ was diluted two and four times and then pretreated, as described in this study. The samples were accurately and precisely quantified after being diluted at 1:2 and 1:4 ratios. The results are described in Table 4. The RSD of all determinations was below 10%, while the determined amounts ranged between 94 to 103% of the nominal concentrations for all analytes. Undoubtedly, the results satisfactorily fulfilled the established criteria [23].

#### 2.2.8. Stability

The stability of two QC samples, QCL and QCH, of each analyte was assessed under different conditions, including three freeze–thaw cycles following storage at −80 °C, autosampler stability at 10 °C for 24 h, short-term stability at room temperature for 24 h, and long-term stability at −80 °C for 30 days. A stability study is considered a curtail step in bioanalytical method validation; the deviation in accuracy and precision of the samples may indicate significant analyte–matrix reactivity or analyte lability. However, the results proved that ABM and TAM, including their metabolites, were stable in the rat plasma. The quantified concentrations remained between 85 and 115% for all analytes under all stability conditions. High and Low QC samples were recruited in the short-term, long-term, and freeze–thaw stability studies, while the final extracts of the QC samples were tested for autosampler stability. The detailed measurements are listed in Table 5.

### 2.3. Application to Pharmacokinetic Study

**Figure 5:**
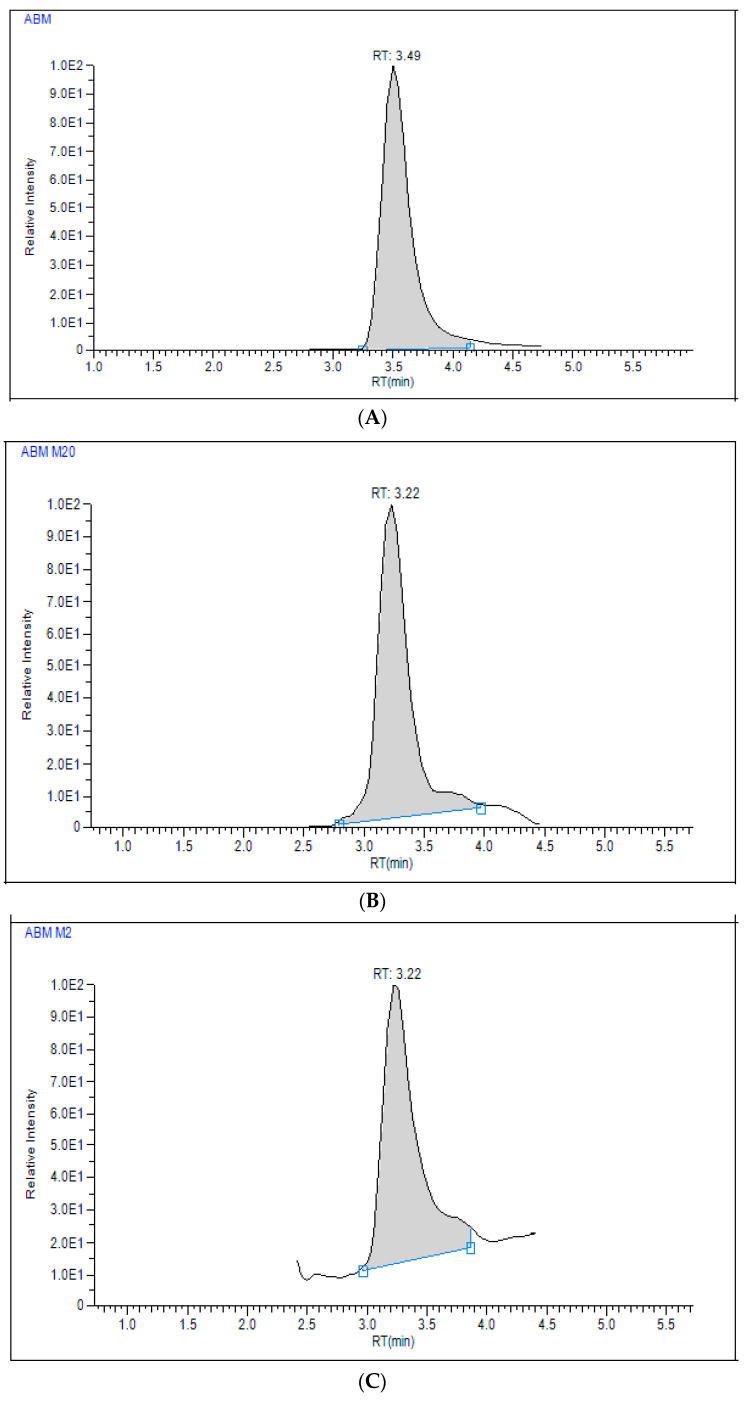

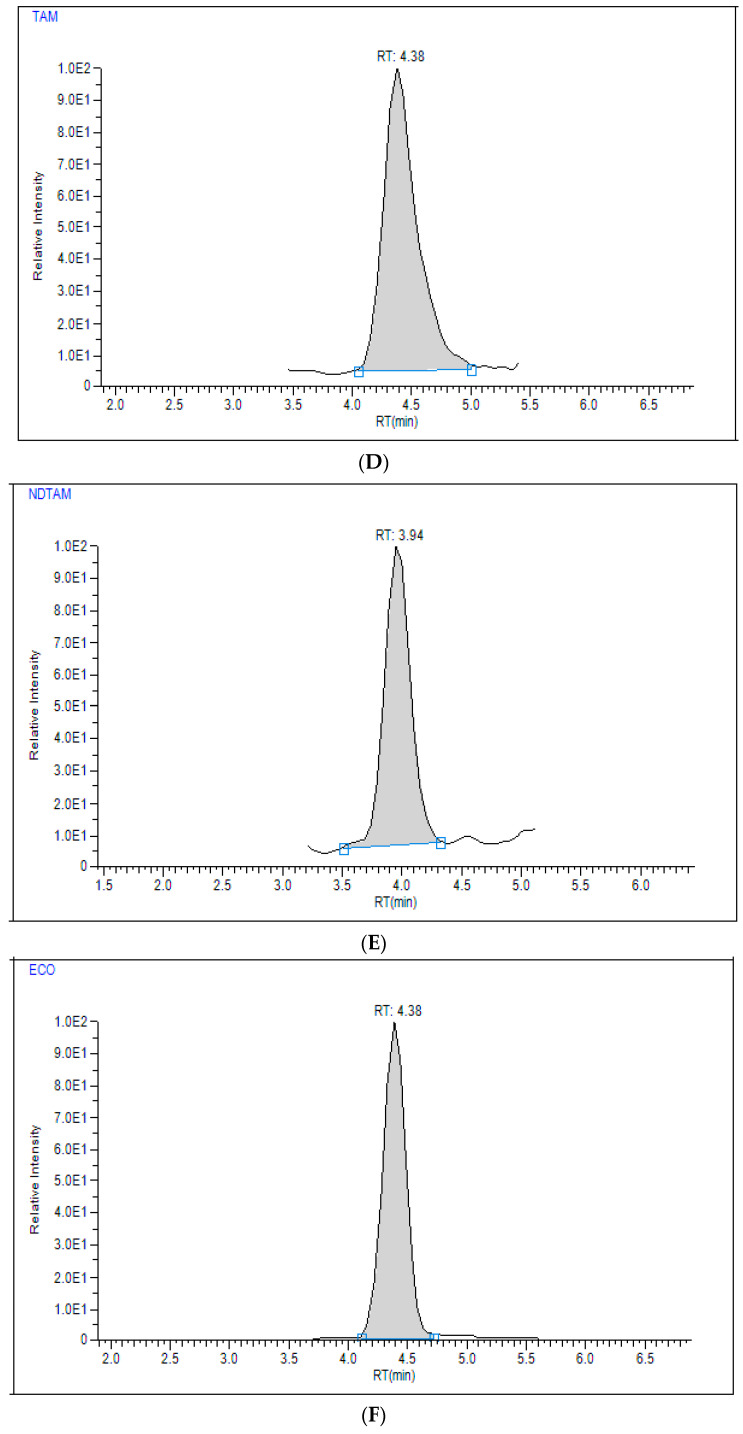
Typical multiple reaction monitoring (MRM) chromatograms for in vivo rat plasma sample of abemaciclib (ABM) (**A**), M2 (**B**), M20 (**C**), tamoxifen (TAM) (**D**), NDTAM (**E**), and IS (**F**) after oral administration of 30 mg/kg ABM and 8 mg/kg TAM.

**Figure 6:**
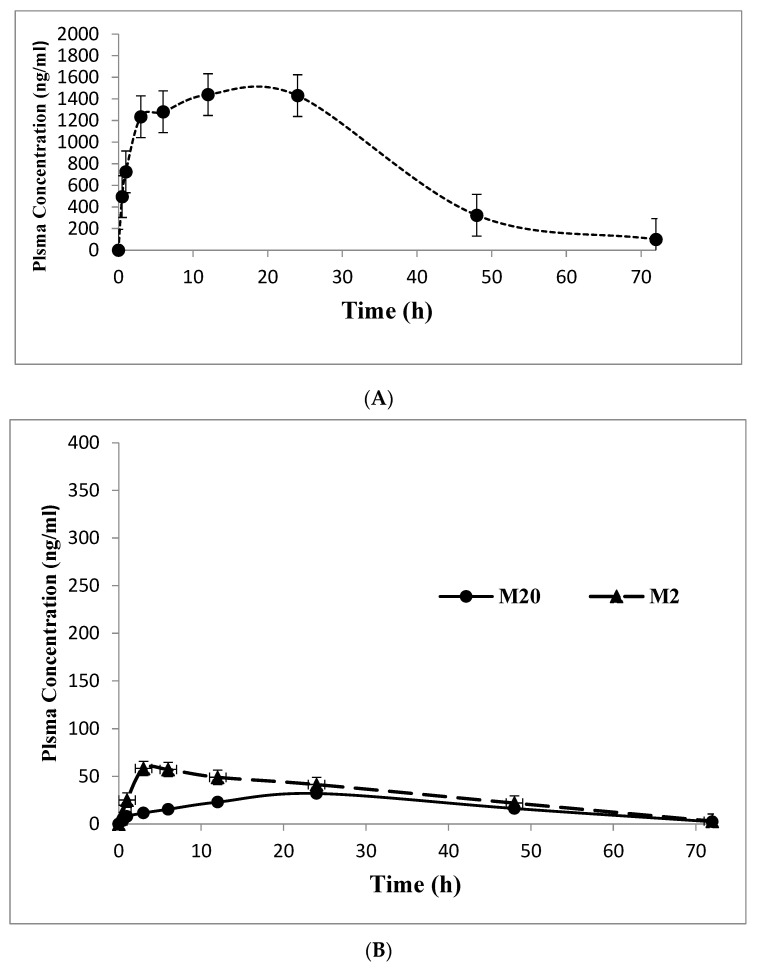
Mean plasma concentration–time profile of abemaciclib (**A**) and its active metabolites M2 and M20 (**B**) in rats after oral administration of 30 mg/kg ABM and 8 mg/kg TAM (n = 6, mean ± SD).

**Figure 7:**
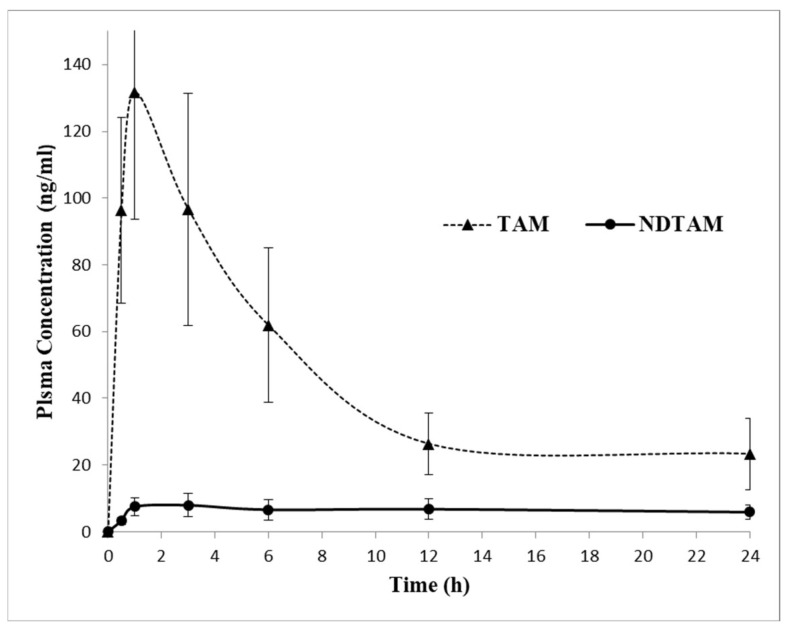
Mean plasma concentration–time profile of tamoxifen (TAM) and its active metabolite NDTAM in rats after oral administration of 30 mg/kg ABM and 8 mg/kg TAM (n = 6, mean ± SD).

The applicability of the developed LC–MS/MS method was demonstrated by measuring ABM, and its active metabolites M2 and M20, and TAM, and its active metabolite NDTAM, in rat plasma samples after oral administration of 30 mg/kg ABM and 8 mg/kg TAM under fasting conditions. The typical MRM chromatograms of rat plasma for ABM, M2, M20, TAM, and NDTAM after oral administration are shown in Figure 5. Furthermore, this method was utilized to support pharmacokinetic studies in rats, where the plasma concentration–time curves and areas under the plasma concentration–time curves (AUC_0–∞_−24 h) of ABM, M2, M20, TAM, and NDTAM are presented in Figure 6 and Figure 7, respectively. All measured samples were within the calibration range for all analytes. The pharmacokinetic parameters of ABM, M2, M20, TAM, and NDTAM from non-compartment model analysis are summarized in Table 6. The C_max_ values (±standard deviation, SD) for ABM, M2, M20, TAM, and NDTAM were 1441 (255), 75 (8), 31 (5), 141 (15.8), and 10 (0.90) ng/mL at the T_max_ of 12, 6, 1, 1, and 1 h, respectively. The AUC_0–t_ values (±SD) for ABM, M2, M20, TAM, and NDTAM were 28,233 (2786), 1021 (113), 1219 (163), 1109 (153.5), and 158 (59) ng/mL·h, respectively. Whereas the AUC_0–∞_ values were 58,514 (5762), 1281 (167), 1409 (233), 1309 (211.3), and 230 (78) ng/mL·h, respectively. The observed CI/Fl values were 0.021, 0.008, 0.007, 0.073, and 0.003 L/h for ABM, M2, M20, TAM, and NDTAM, respectively. The pharmacokinetic results for ABM, M2, M20, TAM, and NDTAM from the current assay closely matched the findings from several publications [12,20,21]. For the M18 samples, measurements were below both the limit of detection (LOD) and the LLOQ. This was related to the time needed for the formation of the metabolites. Whereas M2 and M20 are directly formed from ABM (Figure 1), M18 is formed from these two metabolites, thus involving two metabolic reactions [12]. Alternatively, these low values may be due to M18 being a minor metabolite (13%) or due to inter-individual variations in metabolism among rats. Furthermore, M18 is a known metabolite of abemaciclib in humans and other species, and including it during method validation of the developed assay ensures that the developed LC–MS/MS method is broadly applicable to future preclinical studies or studies in different matrices where M18 may be present at measurable levels. Additionally, incorporating M18 during method validation of the developed assay confirms that the assay is capable of detecting and quantifying this metabolite if it occurs at higher concentrations in other experimental designs, supporting the robustness and comprehensiveness of the developed method.

### 2.4. Comparison with Previously Published Methods

Key parameters of the proposed method and relevant previously published works are shown in Table 7. The bioanalytical UPLC–MS/MS method developed in this work is the first one reported for the simultaneous quantification of ABM and TAM with their major metabolites M2, M18, M20, and NDTAM in rat plasma with pharmacokinetic evaluation. Adequate precision and accuracy of the method in the upper and lower determination ranges were confirmed, as opposed to previously published bioanalytical methods that either did not reported pharmacokinetic characterizations [17], exhibited low extraction recoveries from biological matrices [19], or did not include all analytes under investigation [12,13,14,15,16,17,18,19,20,21]. Moreover, Jacobs et al. [22] developed an LC–HRMS method for the determination of eight anti-breast cancer drugs that have not received FDA approval, including ABM and TAM but without their metabolites, in four biological matrices. This method used a lot of biological samples and did not offer pharmacokinetic characterization. The newly developed and validated UPLC–MS/MS method shows excellent extraction recoveries, sample cleanup, and negligible matrix effects. Additionally, the method incorporates a streamlined sample preparation workflow, rapid chromatographic separation, and minimal solvent consumption. These features facilitate high-throughput analysis of biological samples and enhance its suitability for pharmacokinetic studies.

## 3. Discussion

Developing a single LC–MS/MS method for the simultaneous quantification of abemaciclib (ABM), tamoxifen (TAM), and their active metabolites requires balancing differences in ionization behavior and chromatographic properties across all analytes. In this study, a unified analytical strategy was adopted rather than optimizing each analyte individually, which allowed consistent performance across both ABM and TAM and related metabolites and aligns with previously reported multi-analyte approaches [12,13]. All analytes, including ABM, TAM, and its major metabolites, showed stable responses in positive ionization mode. Optimization of ion source conditions further improved signal quality, particularly for lower-abundance metabolites, while TAM and NDTAM generally exhibited stronger and more consistent responses. This balance in signal behavior contributes to the overall robustness of the method.

Chromatographic separation presented a predictable challenge, especially for ABM and its structurally related metabolites, which tend to exhibit similar retention characteristics. By contrast, TAM and NDTAM were more easily retained and resolved under the optimized conditions. Rather than forcing complete baseline separation, which often comes at the cost of longer run times or compromised peak shape, the method relied on selective SRM transitions to ensure accurate quantification. The use of methanol as the organic modifier, together with ammonium formate, played an important role in improving peak symmetry and maintaining consistent retention across all analytes. Sample preparation was intentionally simplified using protein precipitation with acetonitrile. Despite its simplicity, this approach provided consistent recovery and minimal matrix interference for both ABM and TAM analytes, supporting its suitability for routine and high-throughput analysis. This observation is in line with previously reported methods that favor practical workflows without compromising analytical performance [12,15]. The selection of econazole as an internal standard further supported consistent quantification across all analytes.

The validation results confirmed that the method meets regulatory expectations [23] and performs reliably across the studied concentration ranges. The use of a weighted calibration model (1/x^2^) was particularly important to maintain accuracy at lower concentrations, especially for metabolites. Differences in signal intensity between analytes did not translate into variability in quantification, indicating that the method is sufficiently sensitive for pharmacokinetic applications. Carry-over was primarily observed for the parent compounds ABM and TAM, which is consistent with the lipophilic nature of these two drugs. The implemented mitigation strategies, including extended washing and injection sequence optimization, effectively controlled this issue and allowed reproducible analysis.

The method was successfully applied to a pharmacokinetic study, where the profiles of ABM, TAM, and their major metabolites (M2, M20, and NDTAM) were consistent with previously reported data [12,20]. This agreement supports the accuracy and reliability of the developed assay. Importantly, the ability to monitor both ABM and TAM metabolic pathways within a single run represents a clear advantage over previously reported methods that addressed these analytes separately or incompletely [12,13,14,15,16,17,18,19,20,21]. A limitation of the present study is that validation was performed in rat plasma. Therefore, further validation in human plasma is required before clinical application. Nevertheless, the developed method provides a practical, efficient, and comprehensive tool for preclinical pharmacokinetic studies involving ABM–TAM combination therapy.

## 4. Materials and Methods

### 4.1. Chemical and Reagents

Reference standards of abemaciclib, M2, M18, M20, tamoxifen, and NDTAM (purity > 99.0%) were purchased from MedChemExpress LLC (Monmouth Junction, NJ, USA). Econazole (IS, purity > 97%) was obtained from Sigma-Aldrich (St. Gallen, Switzerland). Ammonium formate and ammonium bicarbonate (LC–MS grade) were supplied by Merck (Darmstadt, Germany). Acetonitrile (Optima™ LC/MS Grade) was obtained from Fisher Chemical™ (Teddington, UK). Methanol hypergrade for LC–MS (LiChrosolv^®^) was obtained from Supelco (Darmstadt, Germany). HPLC-grade water was obtained from an in-house Milli-Q plus purification system purchased from Millipore (Burlington, MA, USA). Drug-free rat plasma was provided from the Animal Care Center at the College of Pharmacy, King Saud University, Saudi Arabia. Rat plasma was used in this study instead of human plasma because there is a significant correlation between the lipoprotein lipid and protein profiles in human and rat plasma [24].

### 4.2. Instrumentation and Chromatographic Conditions

The chromatographic separation was carried out using LC System (Thermo Scientific, Darmstadt, Germany). The LC was coupled with a TSQ mass spectrometer (Thermo Fisher Scientific, Waltham, MA, USA). The ion source was ESI positive ionization mode. The column used was a Kinetex C18 column (100 mm × 2.6 µm, 2.1 mm, Phenomenex, Torrance, CA, USA) protected by a guard column (Phenomenex). The separation was carried out using a gradient elution where mobile phase A consisted of 5 mM ammonium formate in water and mobile phase B consisted of 5 mM ammonium formate in water/methanol (1:9, *v*/*v*). The initial condition was 50% mobile phase B for 0.1 min and then the mobile phase B raised to 100% at 0.8 min. These conditions were maintained until minute 5.5. At minute 5.6, the condition was returned to initial conditions for 0.5 min and then the mobile phase composition shifted to 100% B until 6.7 min. Finally, the conditions were returned to the initial conditions until minute 9. This additional washing step was proven to be adequate for reducing the carry-over of the analytes. The column oven and the autosampler were maintained at 40 and 7 °C, respectively. The injection volume was 5 µL, while the washing solution consisting of methanol/water (1:1) was adequate for washing purposes. The analytes of interest, including the internal standard, were introduced into the mass spectrometer via direct infusion at a constant flow rate of 400 µL/min. The total run time was 4.8 min. ABM, M20, M2, M18, TAM, NDTAM, and IS were ionized via electrospray ionization (ESI) in positive ion mode. The acquisition was performed using multiple reaction monitoring (MRM) of the transitions from protonated precursor ions [M +H]^+^ to particular daughter ions to quantity each compound. Following SRM optimization, the ion source was maintained at 3800 V of spray voltage. The sheath gas was 45 Arb while the auxiliary and sweep gases were 5 and 0 Arb, respectively. The ion transfer tube temperature was 375 °C, while the vaporizer temperature was 400 °C. Full-scan mass spectra were recorded in order to select the most abundant *m*/*z* value. The most abundant precursors were *m*/*z* 507.30 → 393.05 for ABM, *m*/*z* 523.51 → 409.21 for M20, *m*/*z* 479.20 → 393.05 for M2, *m*/*z* 495.20 → 409.05 for M18, *m*/*z* 372.35 → 71.88 for TAM, *m*/*z* 358.10 → 57.90 for NDTAM, and *m*/*z* 381.10 → 125.00 for IS. The postulated fragmentation patterns of these analytes are shown in Figure 2.

### 4.3. Preparation of Stock, Standards, Calibrators, and Quality Control (QC) Samples

Independent stock solutions of all analytes were prepared in dimethyl sulfoxide (DMSO) at 1 mg/mL in duplicate. The working solutions were prepared at different levels, ranging from 50 µg/mL down to 10 ng/mL, which were achieved by diluting an adequate amount of the stock solution in methanol. Econazole (IS) was dissolved in DMSO at 1 mg/mL, which was further diluted with acetonitrile to reach a concentration of 100 ng/mL. This solution was used as precipitation solution as well. Working solutions were 10-fold diluted with control plasma to prepare 8 calibration standards at concentrations of 1, 5, 20, 50, 250, 500, 750, and 1000 ng/mL for ABM, TAM, and M20; 3, 10, 20, 50, 250, 500, 7500, and 1000 ng/mL for M2; 5, 20, 50, 100, 200, 300, 400, and 500 ng/mL for M18; and 1, 5, 10, 20, 50, 200, 400, and 500 ng/mL for NDTAM. In addition, blanks (no analyte spiked, but processed with internal standard) and double blanks (no analyte spiked and no internal standard) were prepared. QC samples containing all analytes were prepared by diluting the working solutions 10-fold in control plasma at the following concentration levels: the lower limit of quantification (LLOQ), low (QCL), mid (QCM), and high (QCH). The final concentrations of ABM, TAM, M20, M2, M18, and NDTAM were, respectively, 1, 3, 5, and 1 ng/mL for the LLOQ; 3, 9, 15, and 1 ng/mL for the QCL; 400, 400, 250, and 250 ng/mL for the QCM; and 800, 800, 400, and 400 ng/mL for the QCH. To obtain each calibration curve for each drug, the peak area ratios to IS were processed. Alternatively, the corresponding regression equation was derived.

### 4.4. Sample Pretreatment

The plasma samples were prepared using the protein precipitation method. The precipitation using acetonitrile was adequate to recover the analyte of interest, including the internal standard. A volume of 50 µL was taken in 1.5 mL Eppendorf tubes, to which 200 µL of internal standard solution was added and vortexed immediately at 3000 RPM. The tubes were centrifuged at 15,000 RPM and room temperature for 15 min. Later, 100 µL of the supernatant was collected in a micro HPLC vial containing 100 µL of 5 mM ammonium formate in water/methanol (1:1). The vial was vortexed to ensure homogeneity of the sample and 5 µL was injected into the LC system.

### 4.5. Pre-Study Validation

In accordance with US FDA guidelines, the UPLC–MS/MS method was fully validated in terms of linearity, accuracy, precision, selectivity, carry-over, recovery, and matrix effect (ME) [23].

#### 4.5.1. Selectivity and Specificity

For evaluation of method selectivity and specificity, six different batches of rat plasma from six individual animals were used. Three independent lots were prepared for each particular plasma batch: the first lot was prepared by spiking the analyte and internal standard at the LLOQ level; second, a blank sample containing only the internal standard; and finally a double blank sample containing neither analytes nor internal standard. No interfering peaks should be observed above 20% of the LLOQ peak area for all analytes of interest. In addition, no interfering peaks should be observed above 5% of the internal standard peak area. The purpose of this step was to evaluate potential interferences from endogenous substances.

#### 4.5.2. Lower Limit of Quantification (LLOQ)

The LOQ was first estimated based on the calibration curve and then verified by the signal-to-noise ratio of the LLOQ. The signal-to-noise ratio should be more than 10. The established LLOQ should be validated according to the acceptance criteria. The accuracy of the level should range from 80 to 120% with a maximum RSD of 20% [23].

#### 4.5.3. Linearity and Calibration Curve

First, the calibration curve was prepared by analyzing the calibration standards that were previously prepared in rat plasma. The data should be collected from at least 3 separate runs each run in duplicate. The calibration curve was constructed by plotting the area ratio of the analyte of interest to the IS against the nominal concentration of the calibration standard on the x-axis, with a weighting factor of 1/x^2^, where x is the analyte concentration. At least 75% of the calibration level of the calibration curve should exhibit a calculated concentration ranging from 85 to 115% of the nominal concentration and 80 to 120% of the LLOQ. At least 50% of the calibrators should meet these criteria for each level [23].

#### 4.5.4. Accuracy and Precision

The accuracy and precision were investigated using 5 determinations at 4 levels of QC plasma samples (LLOQ, QCL, QCM, and QCH) on three different days. Accuracy was calculated for each level within a run (n = 6) and between runs (n = 18) as a percentage of the yielded practical concentration from the nominal concentration. Meanwhile, the precision was expressed as the relative standard deviation (%RSD) of the yielded concentrations for each run. The accuracy was considered acceptable between 85 to 115% and RSD ≤ 15%. However, the LLOQ might show a wider range in accuracy from 80 to 120% and a variation of ≤20% [23]. For all metabolites, the last acceptance criteria were used at all concentration levels.

#### 4.5.5. Matrix Effect (ME) and Recovery

In this study, three different sample types were recruited. First, processed plasma samples were prepared, where the unspiked plasma was processed and treated as described in the sample preparation (Section 4.4). These types of samples were spiked (after treatment) at high- and low-concentration levels. Second, standard solutions were prepared without plasma (matrix less solution). These solutions were prepared in exactly the same manner but without plasma. Lastly, spiked QC samples at low and high levels (Spiked before treatment) were prepared and treated as described in Section 4.4.

The extraction recovery was the yield of the analyte collected after spiked QC plasma samples (spiked before treatment) were prepared using the same protein precipitation method (Section 4.4). The recovery was calculated as a percentage (%) of the measured concentration compared to the measured concentration in the spiked processed plasma samples (spiked after treatment). The matrix effect was studied using six different rat plasma batches. Each batch was pretreated as described in Section 4.4 and spiked at low and high concentrations (spiked after treatment). The matrix factor (MF) was calculated by comparing the area of the compound (analyte of interest or IS) in the spiked plasma extract (spiked after treatment) to the standard solution without plasma (matrix-less). Consequently, the IS normalized matrix factor was calculated by dividing the MF of the analyte by the MF of the IS. The matrix effect was considered significant if the RSD of the IS-normalized MF was >15%. A similar approach was utilized in previous reports [10,13].

#### 4.5.6. Carry-Over

The carry-over was investigated by injecting a double blank sample after the upper limit of quantification (ULOQ). The response of the analyte of interest in the double blank samples should not exceed 20% of the response of the LLOQ of the analyte of interest and not more than 5% of the IS.

#### 4.5.7. Dilution Integrity

The dilution integrity of samples verified the dilution of high levels of samples above the linear range of the method. This was achieved by spiking high levels of analytes in plasma samples. Consequently, the plasma samples were diluted with blank plasma at ratios of 1:2 and 1:4. The samples were pretreated as described in the established method. The percentage of the yielded analytes was compared to the nominal concentration and should be within 85 to 115% and RSD ≤ 15% for ABM and TAM and ±20% for all metabolites (M2, M18, M20, and NDTAM).

#### 4.5.8. Stability

Evaluation of the stability of the plasma samples was carried out at 2 QC levels (LQC and HQC) using three determinations. For short-term stability, plasma samples were kept on the bench for 6 h at room temperature, while for long-term stability, plasma samples were kept in a freezer at −20 °C for 30 days. In addition, three cycles of freeze and thaw (F/T) were conducted to evaluate the impact on stability. Autosampler stability was assessed for 48 h. The samples were considered stable if the sample concentration ranged from 85 to 115% of the nominal concentration [23].

### 4.6. Pharmacokinetic Study

To evaluate method applicability, a well-established pharmacokinetic study was conducted. The animals recruited for the study were 6 healthy male Wistar rats, weighing about 250 ± 25 g, which were obtained from the Animal Care Center of King Saud University, Saudi Arabia. Before the experiment, the rats were given seven days to adjust to their new environments in the lab. Prior to the experiment, food was prohibited for 12 h but water was still freely available. All experiments were performed in accordance with the guidelines of King Saud University’s Institutional Research Ethics Committee (REC) with ethics number KSU-SE-23-81 and approval date 19 October 2023. At the beginning of the study, the rats were given both drugs orally at 30 mg/kg of ABM [25] and 8 mg/kg of TAM [26]. The drugs were homogenized and suspended in 1% methyl cellulose in water (*w*/*v*) to produce a stable mixture. A 300 µL blood sample was collected in a 1.5 mL polythene tube that contained ethylene diamine tetra-acetic acid di-potassium (EDTA K2) as an anticoagulant. The time intervals for sampling were 0.5, 1, 2, 4, 6, 8, 12, 24, 36, 48, and 72 h. Zero-time samples were collected before oral drug administration. Immediately, all collected samples were centrifuged at 3500 rpm for 10 min at 4 °C. The obtained plasma samples were kept at −80 °C. The plasma sample pretreatment was similar to that established in the current study (4.4.). The maximum plasma concentration (C_max_) and the time to reach the maximum concentration (T_max_) were determined from the plasma concentration profile. The area under the curve up to the last measurable time (AUC_0–t_) was determined following the linear trapezoidal rule by summing the area from zero to the last detectable time points. The elimination rate constant (CI/F) was determined from the slope of the linearity curve constructed with the points after C_max_. The area under the curve from zero to infinity (AUC_0–∞_) was determined by adding the last measurable concentration divided by CI/F to AUC_0–t_. The PK calculations were conducted using a noncompartmental analysis (NCA) model with PKSolver add-in software (Version 2.0) [27].

## 5. Conclusions

An UPLC–MS/MS bioanalytical assay for the simultaneous quantification of ABM, TAM, and its metabolites (M2, M18, M20, and NDTAM) in rat plasma was developed and validated. This method demonstrated linearity, accuracy, precision, selectivity, and sensitivity for all six analytes. The suggested assay’s broad calibration curve made it possible to efficiently quantify pharmacokinetic parameters following oral administration of abemaciclib (30 mg/kg) and tamoxifen (8 mg/kg). The current method is distinguished by suitable extraction recovery and the absence of matrix interference. ABM, TAM, and its metabolites M2, M18, M20, and NDTAM were stable in rat plasma under several tested conditions. Furthermore, the applicability of the developed assay was demonstrated in a preclinical pharmacokinetic study in rats. Further validation in human plasma is required for future clinical or therapeutic drug monitoring applications, as the approach was developed in an animal model.

## Figures and Tables

**Table 1:** Regression parameters to determine abemaciclib (ABM) and tamoxifen (TAM) with their metabolites using the developed LC–MS/ MS method.

Parameter	ABM	M20	M2	M18	TAM	NDTAM
Calibration Model	Linear Regression					
Weighting Factor	1/x^2^					
Concentration range (ng/mL)	1–1000	1–1000	3–1000	5–500	1–1000	1–500
Coefficient of determination (R^2^)	0.9991	0.9971	0.9931	0.9974	0.9967	0.9942
Intercept (a)	6.49 × 10^−3^	4.05 × 10^−3^	8.51 × 10^−3^	6.44 × 10^−3^	3.51 × 10^−3^	6.31 × 10^−3^
Slope (b)	3.22 × 10^−3^	4.56 × 10^−4^	4.24 × 10^−3^	2.07 × 10^−3^	8.26 × 10^−4^	4.60 × 10^−4^
LLOD	0.3	0.3	1	1.5	0.3	0.3
LLOQ	1	1	3	5	1	1

**Table 2:** The accuracy and precision data for the determination of abemaciclib (ABM) and tamoxifen (TAM) with their active metabolites in rat plasma.

Analyte		Within-Run (n = 6)			Between-Run (n = 18)		
	Nominal (ng/mL)	Measured (ng/mL)	Accuracy (%)	CV (%)	Measured (ng/mL)	Accuracy (%)	CV (%)
**ABM**	1	0.99	98.91	3.84	1.004	100.4	5.67
	3	2.96	98.79	3.04	3.02	100.71	3.93
	400	405.32	101.33	2.35	411.68	102.92	5.11
	800	826.19	103.27	1.54	813.43	101.68	4.77
**M20**	1	1.02	102.39	4.11	1.015	101.50	6.69
	3	3.02	100.52	3.64	3.24	107.9	5.24
	400	398.86	99.72	2.75	409.38	102.34	5.31
	800	812.57	101.57	3.02	828.11	103.51	4.32
**M2**	3	3.18	106.01	3.89	3.04	101.37	9.42
	9	8.98	99.11	4.67	9.89	102.10	5.30
	400	389.04	97.26	6.76	410.19	102.55	6.28
	800	834.95	104.37	5.76	837.44	104.68	4.03
**M18**	5	5.17	103.35	3.65	5.27	105.39	5.89
	15	14.58	97.18	6.83	14.80	98.66	7.14
	250	256.25	102.50	3.78	258.13	103.25	5.54
	400	404.40	101.10	4.22	409.37	102.34	4.51
**TAM**	1	1.03	102.7	3.72	1.07	107.2	7.25
	3	3.16	105.28	4.65	3.14	104.65	5.51
	400	385.69	96.42	5.00	394.65	98.66	5.89
	800	777.76	97.22	4.64	789.92	98.74	4.27
**NDTAM**	1	0.99	99.16	6.54	0.979	97.90	7.57
	3	2.89	96.45	5.72	2.87	95.85	6.66
	250	243.43	97.37	2.87	242.85	97.14	5.50
	400	390.07	97.52	4.63	385.11	96.28	6.41

**Table 3:** Extraction recovery for the analysis of of abemaciclib (ABM) and tamoxifen (TAM) with their active metabolites in rat plasma using the developed LC–MS/MS method.

		Recovery		Normalized Matrix Factor	
Analyte	QC Level (ng/mL)	(%)	RSD	%	RSD
ABM	3	92.39	5.31	0.99	7.37
	800	93.67	5.5	0.95	11.5
M20	3	94.37	8.95	1.01	7.22
	800	92.00	4.70	0.97	8.50
M2	9	91.00	9.01	1.00	11.73
	800	90.14	7.10	0.92	12.9
M18	15	107.78	6.70	0.90	10.91
	400	102.59	7.80	1.08	4.30
TAM	3	95.36	9.69	1.03	11.32
	800	91.01	4.40	0.87	3.50
NDTAM	3	106.64	9.30	1.02	11.20
	400	100.90	3.40	0.94	10.50
IS	1	92.15	9.41	0.92	10.35

**Table 4:** Evaluation of the dilution integrity of abemaciclib (ABM) and tamoxifen (TAM) with their active metabolites in rat plasma using the developed LC–MS/MS method.

	Spiking Concentration	Dilution Fold (1:2)		Dilution Fold (1:4)	
Analytes	ng/mL	Recovery (%)	RSD	Recovery (%)	RSD
ABM	2000	100.43	4.79	104.78	4.39
M20	2000	97.92	2.06	94.41	5.27
M2	2000	95.68	0.27	104.83	0.15
M18	1000	98.28	1.0	93.72	2.04
TAM	2000	93.35	1.60	101.10	2.31
NDTAM	1000	102.75	9.34	100.45	8.46

**Table 5:** Stability results for abemaciclib (ABM) and tamoxifen (TAM) with their active metabolites in rat plasma under different conditions.

		Autosampler Stability at 10 °C (24 h)		Short Term Stability at Room Temperature (24 h)		Long Term Stability at −80 °C (30 Days)		Freeze and Thaw Stability at −80 °C (3 Cycles)	
Analyte		Recovery (%)	RSD	Recovery (%)	RSD	Recovery (%)	RSD	Recovery (%)	RSD
ABM	3	98.03	6.12	97.67	7.88	96.74	6.17	101.77	4.54
	800	102.09	7.30	98.25	2.21	95.05	6.11	102.75	6.21
M20	3	103.17	5.48	97.14	4.01	92.66	4.73	97.52	7.13
	800	97.24	6.10	94.31	2.02	97.47	5.22	97.43	4.40
M2	9	98.58	8.22	94.68	8.45	94.64	6.10	96.79	8.48
	800	96.37	5.31	93.81	3.50	93.93	6.62	102.05	7.21
M18	15	100.26	7.15	106.50	6.87	95.08	5.80	102.36	6.45
	400	102.85	2.51	94.49	3.71	100.92	4.42	100.32	3.40
TAM	3	104.44	5.65	98.95	5.90	94.99	4.91	95.52	7.76
	800	99.28	2.70	97.78	4.02	90.27	4.03	94.16	6.54
NDTAM	3	97.50	4.23	93.23	8.63	92.92	5.41	97.63	3.01
	400	103.18	1.32	104.12	5.8	91.26	3.62	98.59	8.93

**Table 6:** The pharmacokinetic parameters of abemaciclib (ABM), its active metabolites M2 and M20, tamoxifen (TAM), and its active metabolite NDTAM in rat plasma after oral administration of 30 mg/kg ABM and 8 mg/kg TAM (n = 5, Mean ± SD).

Parameters	Unit	ABM	M2	M20	TAM	NDTAM
AUC_0–t_ ^a^	ng/mL·h	28,233 ± 2786	1021 ± 113	1219 ± 163	1109 ± 153.5	158 ± 59
AUC_0–∞_ ^b^	ng/mL·h	58,514 ± 5762	1281 ± 167	1409 ± 233	1309 ± 211.3	230 ± 78
C_max_ ^c^	ng/mL	1441 ± 255	75 ± 8	31 ± 5	131 ± 15.8	10 ± 0.9
T_max_ ^d^	h	12	6	1	1	3
Cl/F ^e^	L/h	0.0210	0.0081	0.0076	0.0729	0.0030
t_1/2_ ^f^	h	31 ± 4.2	12 ± 2.1	ND	10 ± 1.6	12 ± 3.6

^a^ Area under the curve up to the last sampling time; ^b^ Area under the curve extrapolated to infinity; ^c^ Maximum plasma concentration; ^d^ Time taken to reach the maximum plasma concentration; ^e^ Total clearance of drug from plasma after oral administration; ^f^ Half-life.

**Table 7:** Comparison of the proposed LC–MS/MS method with previously published methods.

Reference	Analytes	Analytical Technique	Biological Matrices	Sample Preparation Technique	Mean Extraction Recovery	Linear Range
Martínez-Chávez et al., 2021 [12]	ABM, M2, M18, M20	UHPLC–MS/MS	Human and mouse plasma	PPT w/ACN	90–105%	1–600 ng/mL ABM, 0.5–300 ng/mL M2, M20, 0.2–120 ng/mL M18
Martínez-Chávez et al., 2019 [13]	ABM, PAL, RIB	LC–MS/MS	Human and mouse plasma	PPT w/ACN	79–83%	2–200 ng/mL
Habler et al., 2023 [14]	ABM, PAL, RIB, M2, M20	LC–MS/MS	Human serum	PPT w/ACN	89–116%	20–800 ng/mL ABM, 10–400 ng/mL PAL, M2 100–4000 ng/mL RIB, 15–600 ng/mL M20
Margaryan et al., 2022 [15]	ABM, LY3214996 M2, M20	LC–MS/MS	Human plasma, brain tumor	PPT w/MeOH	86–99%	0.2–500 nM
Turković et al., 2022 [16]	PAL, RIB, ABE, ANA, LET, FUL	LC–MS/MS	Human plasma	PPT w/ACN	n.a.	25–5000 ng/mL RIB, 15–3000 ng/mL ABE, 5–500 ng/mL PAL, 1–200 ng/mL ANA, 2.5–500 ng/mL LET, 50–1000 ng/mL FUL
Binkhorst et al., 2011 [17]	TAM, NDTAM, HOTAM, END	LC–MS/MS	Human plasma	PPT w/ACN	64–95%	5–800 nM TAM, NDTAM 0.5–80 nM HOTAM, END
Rama Raiu et al., 2015 [18]	TAM, HOTAM, CEN, 7-DMC	LC–MS/MS	Rat plasma	LLE w/Hex	80–92%	2–200 ng/mL
Antunes et al., 2015 [19]	TAM, NDTAM, HOTAM, END	LC–MS/MS	Dried Blood Spots	EXT w/ MeOH	40–92%	7.5–210 ng/mL TAM, 15–450 ng/mL NDTAM, 0.5–18 ng/mL HOTAM, 1–30 ng/mL END
Shin & Choi et al., 2009 [20]	TAM, HOTAM	HPLC–FLU	Rat plasma	PPT w/ACN	n.a.	n.a.
Williams et al., 2006 [21]	TAM, NDTAM, HOTAM, END	LC–MS/MS	Rat serum	PPT w/ACN	70–99%	0.01–1 µM
This study	ABM, M2, M18, M20, TAM, NDTAM	LC–MS/MS	Rat plasma	PPT w/ACN	91–107%	1–1000 ng/mL ABM, TAM, M20, 3–1000 ng/mL M2, 5–500 ng/mL M18, 1–500 ng/mL NDTAM

Abbreviations: ABE: abemaciclib; PAL: palbociclib; RIB: ribociclib; ANA: anastrazole; LET: letrozole; FUL: fulvestrant; TAM: tamoxifen; M2: N-desethylabemaciclib; M18: hydroxy-N-desethylabemaciclib; M20: hydroxyabemaciclib; NDTAM: N-desmethyltamoxifen; HOTAM: hydroxytamoxifen; END: endoxifen; CEN: centchroman; 7-DMC: 7-desethyl centchroman; PPT: protein precipitation; LLE: liquid-liquid extraction; EXT: extraction; ACN: acetonitrile; MeOH: methanol; Hex: hexane; w/: with; n.a.: not available.

## Data Availability

The original contributions presented in this study are included in the article. Further inquiries can be directed to the corresponding author.
